# [Corrigendum] ENO2 affects the EMT process of renal cell carcinoma and participates in the regulation of the immune micro­environment

**DOI:** 10.3892/or.2026.9135

**Published:** 2026-05-13

**Authors:** Wei-Jie Chen, Wei Yang, Min Gong, Yi He, Da Xu, Jia-Xin Chen, Wen-Jin Chen, Wen-Yan Li, Yu-Qi Wang, Ke-Qin Dong, Xu Song, Xiu-Wu Pan, Xin-Gang Cui

Oncol Rep 49: 33, 2023; DOI: 10.3892/or.2022.8470

Following the publication of this article, the authors have brought to the Editor's attention that they made an error in the compilation of the data in Fig. 4, as it appeared on p. 9. Specifically, some of the blots included for the 769-P cell line experiments in Fig. 4B had inadvertently been included in the figure incorrectly.

The corrected version of Fig. 4, now showing all the correct western blot data in Fig. 4B, is shown on the next page. The authors sincerely apologize for the errors that were introduced during the preparation of this figure, and are grateful to the Editor of *Oncology Reports* for granting them the opportunity to publish a Corrigendum. All the authors agree with the publication of this Corrigendum, and they also regret any inconvenience that this mistake may have caused.

## Figures and Tables

**Figure 4.**
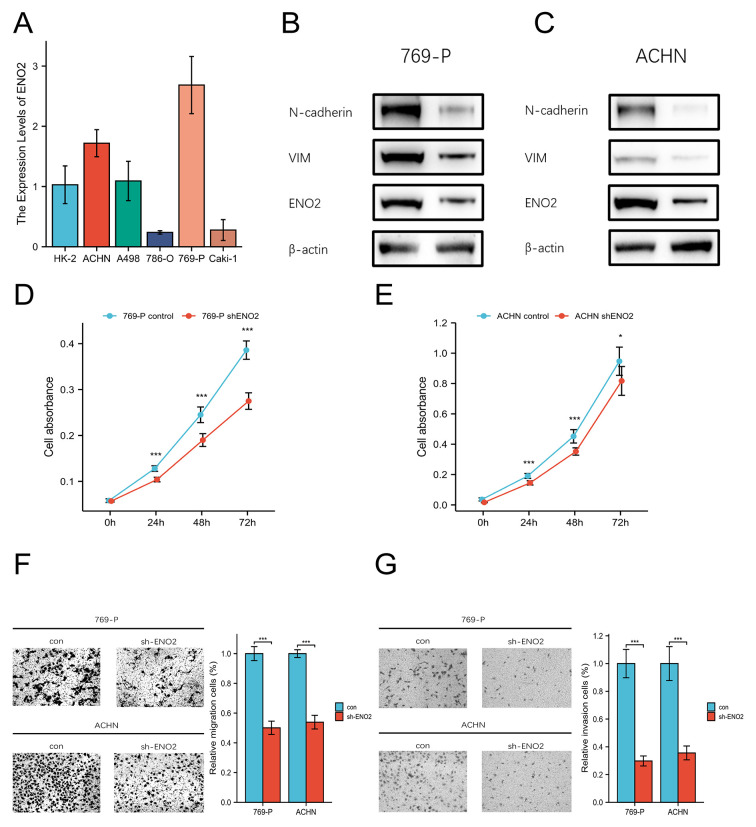
*In vitro* experiments demonstrating that ENO2 knockdown inhibits EMT and ccRCC progression. (A) The mRNA expression levels of ENO2 in HK2 and RCC cells (Caki-1, ACHN, A498, 786-O and 769-P) were detected using reverse transcription-quantitative PCR analysis. (B and C) Western blotting demonstrated that ENO2 expression was effective2ly knocked down in (B) 769-P and (C) ACHN cells. The altered expression levels of the proteins VIM and N-cadherin suggested that ENO2 knockdown was able to prevent the EMT process of RCC. (D and E) Proliferation of (D) 769-P and (E) ACHN cells transfected with control and shENO2 vectors was determined using Cell Counting Kit-8 assays. (F and G) Two sets of RCC cells (control and shENO2) were tested for their capacity to (A) migrate using Transwell assay analysis and (B) invade using Matrigel assay analysis. Scale bar, 100 µm. *P<0.05 and ***P<0.001. ENO2, enolase 2; EMT, epithelial-mesenchymal transition; ccRCC, clear cell renal cell carcinoma; VIM, vimentin..

